# Disrupted Social Hierarchy in Prenatally Valproate-Exposed Autistic-Like Rats

**DOI:** 10.3389/fnbeh.2019.00295

**Published:** 2020-01-15

**Authors:** Péter Pelsőczi, Kristóf Kelemen, Cecília Csölle, Gábor Nagy, Balázs Lendvai, Viktor Román, György Lévay

**Affiliations:** ^1^Laboratory of Cognitive Pharmacology, Division of Pharmacology and Drug Safety, Gedeon Richter Plc., Budapest, Hungary; ^2^Faculty of Pharmaceutical Sciences, Semmelweis University School of PhD Studies, Budapest, Hungary; ^3^Laboratory of Neurodevelopmental Biology, Division of Pharmacology and Drug Safety, Gedeon Richter Plc., Budapest, Hungary; ^4^Division of Pharmacology and Drug Safety, Gedeon Richter Plc., Budapest, Hungary; ^5^Department of Morphology and Physiology, Faculty of Health Sciences, Semmelweis University, Budapest, Hungary

**Keywords:** rats, valproate, autism spectrum disorder, exploratory behavior, hierarchy, prenatal exposure delayed effects, polydipsia, psychogenic

## Abstract

Autism spectrum disorder (ASD) is characterized by impaired socio-communicational function, repetitive and restricted behaviors. Valproic acid (VPA) was reported to increase the prevalence of ASD in humans as a consequence of its use during pregnancy. VPA treatment also induces autistic-like behaviors in the offspring of rats after prenatal exposure; hence it is a preclinical disease model with high translational value. In the present study, our aim was to characterize ASD relevant behaviors of socially housed, individually identified male rats in automated home cages. The natural behavior of rats was assessed by monitoring their visits to drinking bottles in an environment without human influence aiming at reducing interventional stress. Although rodents normally tend to explore their new environment, prenatally VPA-treated rats showed a drastic impairment in initial and long-term exploratory behavior throughout their stay in the automated cage. Furthermore, VPA rats displayed psychogenic polydipsia (PPD) as well as altered circadian activity. In the competitive situation of strict water deprivation controls switched to an uneven resource sharing and only a few dominant animals had access to water. In VPA animals similar hierarchy-related changes were completely absent. While the control rats secured their chance to drink with frequent reentering visits, thereby “guarding” the water resource, VPA animals did not switch to uneven sharing and displayed no evidence of guarding behavior.

## Introduction

Autism spectrum disorder (ASD) is a neurodevelopmental condition characterized by social and communicative impairments and excessive repetitive behaviors (American Psychiatric Association, 2013). Although the pathogenesis of ASD is not fully understood, several factors have been identified as possible contributors such as genetic (Sebat et al., 2007; Klei et al., 2012; De Rubeis et al., 2014) or environmental factors (Brown, 2012; Hertz-Picciotto et al., 2018). Hallmarks of autistic-like symptoms in rodents can be measured with several behavioral assays such as the three-chamber social interaction, self-grooming, ultrasonic vocalization, tube dominance or social playtests. However, in most of these tests the subject is removed from its home-cage and must adapt to a novel environment. In these situations, animals are exposed to excessive human handling that can cause unnecessary stress and might have a serious impact on the natural behavior of the experimental animals. This stress can lead to an elevated level of anxiety which could introduce a strong bias in the results.

With automated home-cages, the experimenter can reduce human interference to a minimum. In automated home-cages animals are kept in their familiar environment, while the social structure of the group is intact for the entire duration of the experiment. This virtually undisturbed environment could reveal more natural spontaneous behaviors that in turn may result in more sensitive assessment methods compared to traditional behavioral assays. Because in ASD social behavior and structure are so crucial, we focused our research interest more on group dynamics rather than only on the individual behavior of subjects.

Prenatal exposure to valproic acid (VPA), a frequently used anticonvulsant medication (Löscher, 2002), is a major non-genetic risk factor of ASD (Bromley et al., 2013; Christensen et al., 2013). VPA treatment also induces autistic-like behaviors in the offspring after prenatal exposure in rats (Schneider and Przewłocki, 2005), including impaired social interaction, excessive repetitive behaviors (Kim et al., 2013; Roullet et al., 2013; Dai et al., 2018; Nicolini and Fahnestock, 2018), reduced ultrasonic communication (Gandal et al., 2010) as well as increased anxiety (Mehta et al., 2011). The well-grounded etiopathology of the model and its symptomatic similarity to the human condition confer this animal model of ASD a high translational value.

Although it is a widely accepted model of ASD, the behavior of groups of prenatally VPA-exposed animals has not been investigated extensively. As described above, automated home-cages offer an excellent means to study non-apparent/underlying behaviors, hierarchy and group dynamics in socially-kept rodents, by avoiding rats even recognize that they are under surveillance. Given the scarcity of studies investigating autistic-like rodents in an automated environment, here we aim at studying the behavior of communities of autistic-like and control rats with as little human intervention possible, besides trying to minimize any stress or subjective bias.

## Materials and Methods

### Prenatal Valproate Treatment

Timed-pregnant Wistar rats (outbred stock, Janvier, France) kept on soy-free diet (Teklad soy protein-free rodent diet, ENVIGO, Madison, WI, USA) and tap water received a single dose of 300 mg/kg sodium valproate (VPA, cat. P4543-10G, Sigma, UK) intraperitoneally in a volume of 2.5 ml/kg physiological saline on gestational day 12. The pregnant rats were transported from Janvier to our animal facility. Control dams received an injection of physiological saline of identical volume at the same gestational time-point. The size of litter was adjusted to 10 for each dam (by removing female pups) and then left undisturbed until the time of weaning on postnatal day 21 when the male offsprings were housed in groups of three or four until behavioral testing.

### Subjects and Husbandry

Twenty 11-week-old male Wistar rats were chosen from the F1 generation (*n* = 10 in control and *n* = 10 in the VPA group, selected from four litters in each treatment group) and were placed separately in two IntelliCages. Animals were implanted with microchips (UNO PICO-ID ISO transponder, UNO BV, Netherlands) under isoflurane anesthesia 1 week prior to the experiments. Rats were kept in the animal facility with a 12 h light/dark cycle (lights off at 4 p.m.), while ambient room temperature was maintained at 22 ± 2°C and 40–50% relative humidity. Food was freely available; water access was related to specific tasks. All efforts were made to minimize the suffering of experimental animals. Experimental procedures were reviewed and approved by the Local Animal Care and Use Committee (PE/EA/2885-6/2016) and were carried out in accordance with the European Animal Protection Directives (Directive 2010/63/EU).

### The IntelliCage Apparatus

The IntelliCage system (TSE Systems, Bad Homburg, Germany^1^) allowed group-housed rats to be assessed for spontaneous behavior and various other behavioral tasks. The size of the central arena was 100 × 100 × 35 cm. As bedding material wood shavings were used (OSAFE, J. Rettenmeier and Söhne GmbH, Rosenberg, Germany). In order to enrich the environment, two black plastic shelters were placed in each cage, allowing the animals to hide and climb (TSE Systems, Bad Homburg, Germany). The IntelliCage has four recording corners. Water was only available in the corners behind remotely controlled doors. When a rat entered a corner, an antenna detected its unique transponder and recorded its visit. It needs to be emphasized that the corner design allowed the entry of only one animal at a time. Each corner housed two drinking bottles, while the left and right sides could be distinguished. The activity of the rats within the corners was monitored by using tracking software (IntelliCage Plus 3.1.1.0, TSE Systems). The principal parameters were: the number of visits to the corners, initiated nose pokes, lick numbers and the duration of all these parameters.

### Training Phases in the IntelliCage

**Figure 1:**
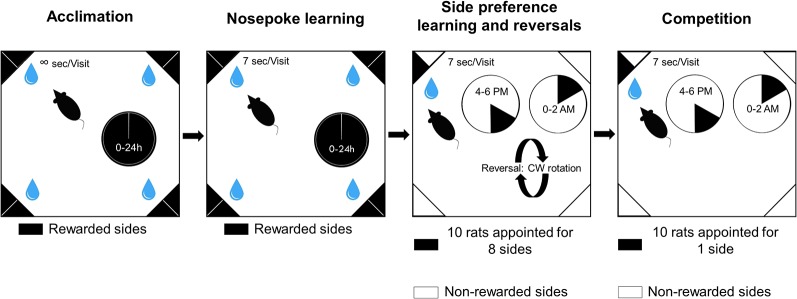
Schematic representation of the experimental phases and visual description of the tasks.

Control and VPA groups were tested in IntelliCages for 41 days. Animals were challenged with gradually more and more complex tasks. The time spent in the IntelliCages was divided into four phases: acclimation, nose poke learning, place preference learning and competition (Figure 1).

In the first phase of the study, rats could habituate to the new environment for 3 days (acclimation). They were allowed to visit any corner any time and choose any bottle to drink from throughout the day. They could also drink from the bottles ad libitum. The number of trials was not limited, thus rats voluntarily visited the corners. The second phase of the study was nose poke learning for 20 days when rats had to initiate trials with a nose poke to gain access to the water bottles for 7 s. The third phase of the study was to train the animals to develop a side preference for an appointed corner in order to study learning behavior for 15 days. One side was rewarded by providing access to water for 7 s, whereas the remaining seven sides did not open after a nose poke. Nose pokes at the seven remaining sides were recorded as incorrect choices. Ten rats were allotted to eight sides. Two rats were allotted to bottles 1 and 2, and one rat to each of the remaining bottles. Reversals were achieved with randomly changing the position of the correct corner to another corner (excluding the current one), and the side was interchanged as well. The reversal was carried out on day 3, 7 and 11. The last phase of the study included the competition task. In this phase, all ten rats were assigned to only one corner in both groups. In, side preference and competition water access was available only for two periods of 2 h each day (4:00–6:00 pm and 0:00–02:00 am). During any visit only the first nose poke resulted in door opening.

### Collection of Blood Samples

Following decapitation, trunk blood was collected rapidly into 0.5 ml plastic tubes and put on ice. Tubes were then centrifuged at 10,000 rpm for 2 min at room temperature. The serum was separated and divided into aliquots of ~300 μl and stored at −80°C. Serum samples (*n* = 6 for each group) were analyzed using a Beckman Coulter AU480 Chemistry Analyzer instrument (Beckman Coulter, Inc., Brea, CA, USA).

### Gene Expression Assay

Each removed tissue sample (*n* = 10, hippocampus (HPC), cerebellum, prefrontal cortex, thalamus) was immersed in RNA *Later* and stored at 4°C overnight, then stored at −20°C. The tissue was homogenized and RNA was extracted using an RNeasy mini kit (Qiagen, Crawley, UK) according to the manufacturer’s protocol. The RNA was stored at −80°C in RNase/DNase-free water. All RNA preparations were analyzed on an Agilent 2,100 Bioanalyzer (Agilent Technologies, Berkshire, UK) to determine the RNA concentration and the quality of the RNA using the RNA integrity number (RIN). cDNA was synthesized from total 1 μg RNA in a 20-μl reaction mixture by using a Superscript VILO cDNA Synthesis kit (Invitrogen) according to the manufacturer’s protocol. Quantitative PCR was carried out using the Applied Biosystems (Carlsbad, CA, USA) Quantstudio 12K Flex Real-Time PCR System, according to the manufacturer’s instructions. Primers and probes for quantitative PCR were purchased from Thermo Fisher Scientific, Waltham, MA, USA (Cry1: Rn01503063_m1; Per1: 01325256_m1; Npas2: Rn01438223_m1; Arntl: Rn00577590_m1; Clock: Rn00573120_m1; Mtnr1a: Rn01488022_m1; b-actin (ACTB), 4352340E). The cycle conditions for quantitative PCR were 95°C for 20 s followed by 40 cycles of 95°C for 1 s and 60°C for 20 s All data were normalized to ACTB expression. Data were calculated using the 2^−ΔΔCT^ method. RQ mean values are normalized to 1 for the control.

### Spontaneous Locomotor Activity

Spontaneous locomotor activity was measured in male rats (*n* = 10 in each group) at postnatal days 26–28 by a six-channel activity monitor manufactured by Experimetria (Hungary). The apparatus consisted of acrylic cages (48.5 cm × 48.5 cm × 40 cm) equipped with 2 × 30 pairs of photocells along the bottom axis of the cage. Additional arrays of photocells (30 pairs) were placed along two opposite sides of the cage at different heights (6.5, 12, 18 and 23 cm) in order to detect rearing responses. The photocell beam, when broken, signaled a count which was then recorded by a computer. The signals were processed by a motion analyzing software that determined the spatial position of the animal with 1 Hz sampling frequency and computed the distance traveled and the time spent by the rats with ambulation, local movement (e.g., grooming), immobility, rearing, etc. Animals were individually placed in the photocell cages; horizontal movements (ambulation time), as well as vertical rearings, were determined for 1 h. Data are expressed as means ± SEM.

### Juvenile Social Play

Pinning as the most characteristic parameter of social play behavior was scored for each pair of male rats (*n* = 10 in each group) on postnatal days 33–36. The testing arena of juvenile social play was a plexiglass cage (42 × 42 × 32 cm) with approximately 2 cm of wood shavings covering the floor. Pairs of rats (from the same treatment group) were assigned for social interaction by using unfamiliar partners (i.e., not a cage mate or littermate). Animals in a test pair did not differ more than 10 g in body weight. On the postnatal day 34 and 35, each animal was introduced to the testing arena for a period of 5 min individually. On the third day (postnatal day 36), the motivation for the play was enhanced by isolating the animals for 4 h before the test. Animals that had been unfamiliar to each other were placed simultaneously into the opposite corners of the previously discovered arena and their behavior was recorded for 15 min. Behavioral elements were assessed using the Observer 5.1 software (Noldus Information Technology B.V., Netherlands). The frequency of pinning as the most characteristic parameter of social play behavior was scored for each pair of animals and expressed as means ± SEM (Panksepp et al., 1984; Trezza et al., 2010). Data are expressed as means ± SEM.

### Maternal Deprivation-Induced Ultrasonic Vocalization

Impairments in communication between pups and their mothers were measured by recording ultrasonic vocalizations. To induce calls, pups (*n* = 10 in each group) were separated from their mothers and placed individually into a cage for 10 min, while calls were being recorded with bat microphones. Calls were digitized with an audio filter and ultrasonic vocalization was recorded and quantified with SonoTrack software (Metris BV., Netherlands). Vocalization was measured at age of 12 days for 10 min. Statistical analysis included the Kruskal–Wallis non-parametric test and the *post hoc* Dunn test. Data are presented as means ± SEM of USV calls count/10 min.

### Von Frey test

Von Frey test was used for estimating paw withdrawal thresholds (expressed in grams) with a series of filaments, that uses a constant number of five stimuli per test. It was conducted with the simplified up-down method as previously described (Bonin et al., 2014; *n* = 10 in each group).

### Statistical Analysis

Exploratory visits (i.e., visits without nose poke or drinking) were aggregated for each day of the experiment by subjects. Generalized linear models (GLM) with log link using negative binomial distribution were constructed. Differences in initial exploratory activity were also compared between groups. To show how exploratory activity changed during the day, visits were aggregated for each 4-h period. For nose poke learning, a cosinor analysis was conducted to see how daytime exploratory activity patterns differed between groups (Cornelissen, 2014). Two main estimates were considered: mesor (mean activity) and amplitude (difference of peak and midline activity). Mean values for groups were compared using the *t*-test. The daytime mean activity was calculated for the first 72 h, groups were compared using bootstrapped Watson’s test.

Drinking volume was estimated by assessing the number of licks and lick duration. For acclimation, the number of licks for each animal was calculated for 4-h periods which also revealed how drinking behavior changed over the course of the day. When competing, animals were restricted both in their access to water bottles, and maximum lick duration for each visit. Therefore, the mean lick duration was calculated for each day of the experiment. In addition, linear mixed-effects models were used to compare groups with subjects as random factors (Bolker et al., 2009). Acclimation was treated separately from the other phases because of the difference in underlying data distribution due to the time limit introduced after acclimation.

In, side preference learning, the proportion of correct nose pokes to all nose pokes was calculated. This response variable was put in a binomial generalized mixed-effects model (GLMM) with treatment as the fixed effect. Binomial distribution was used because the number of correct responses out of all trials was measured. The proportion of correct nose-pokes is expected to change during time due to the initial period and the reversals. Therefore, the drink session was included as a random factor within the model. As dispersion was high, cumulative lick duration was included as a random factor. Differences in drinking volume changed the proportion of visits to the correct corner that was not related to learning. This addition to the model dropped the dispersion to near 1, while the mean of all random factors was close to 0 (−0.02). Type II Wald chi-square test was used to assess the treatment effect. The hierarchy was estimated by differences in lick duration within groups. Total lick duration was calculated for each day of the experiment and evenness of the values was used as a community measurement. Evenness is most often used to describe the distribution of individuals within a community using Pielou’s evenness index ranging from 0 to 1. This measure was adapted to reflect evenness in drinking among individuals, because, the lower the evenness, the stronger the hierarchy that is expected in the community. Hierarchy could be best observed during competition; therefore, rank abundance curves were fitted to the cumulative lick number per hour values of each subject of the groups. Models were compared using Akaike information criterion (AIC) to find the shape of the best fitting model.

Reentering visits (“guarding”) were defined as visits after which the same subject entered the corner. The number of reentering visits was calculated for each subject and day of the experiment. The maximum divided by mean values for groups was calculated to express the distribution of reentering visits within the groups. Experimental phases were merged based on whether water access was unlimited (acclimation, nose-poke learning) or limited (side preference learning, competition) during the given phase. Population-level values were compared by using a linear model. Calculations were carried out by using R^2^.

Statistical evaluation was performed by unpaired *t*-tests to analyze spontaneous locomotor activity (ambulation and rearing), von Frey test, ultrasonic vocalization, and juvenile social play (pinning) results in GraphPad Prism version 7.04 for Windows (GraphPad Software, La Jolla, CA, USA).

## Results

### VPA Rats Show Autistic Phenotype

Preceding studies confirmed autistic behavior of the VPA group (Supplementary Figure S4). In spontaneous locomotor activity they showed impaired rearing (*p* < 0.001), whereas ambulation was unaffected by the treatment. Pinning remained also unchanged. In ultrasonic vocalization (*p* < 0.05) and von Frey test (*p* < 0.001) VPA rats showed significant impairment compared to the control group.

### Decreased Initial Exploration in VPA Rats

**Figure 2:**
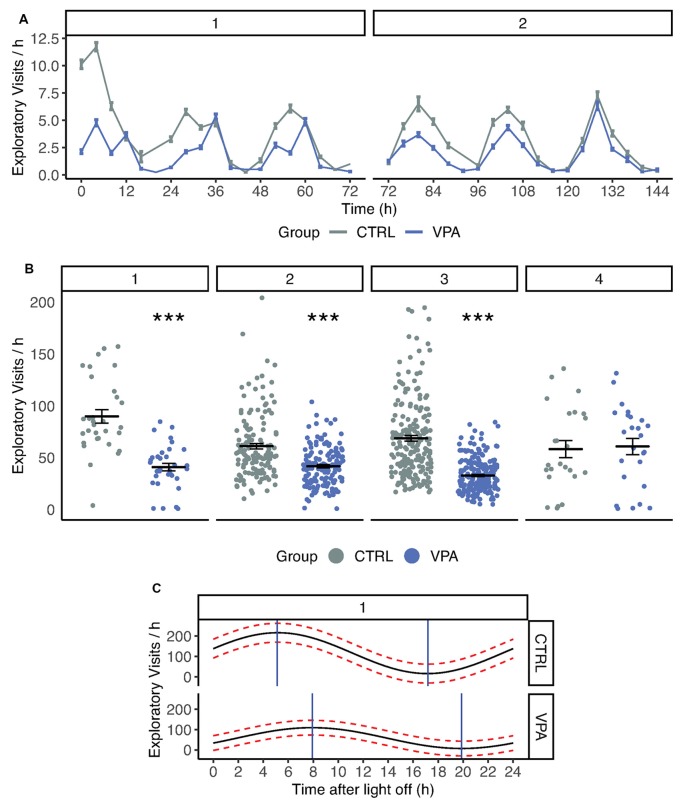
**(A)** Number of exploratory visits in control and VPA groups. 1, 2, indicate phases: Acclimation, Nosepoke learning. Time scale of exploratory visits summed in four-hour periods. Means (±SEM) are shown (*p* < 0.001). **(B)** Number of exploratory visits in control and VPA group. 1, 2, 3, 4 indicate phases: Acclimation, Nosepoke learning, Side preference learning, Competition. In acclimation, nosepoke learning and side preference learning VPA group showed a significant decrease in explorative visits (*p* < 0.001). **(C)** Cosinor analysis of circadian rhythm during acclimation. Mean (black line) and confidence interval (red dotted lines) are drawn for pooled group data. Time after light off values associated with maximum and minimum activity are shown with blue lines. ***p ≤ 0.001.

The exploratory activity was assessed by calculating the number of exploratory visits defined as visits without nose-poke and lick. Control rats tended to explore the novel environment of the IntelliCage, represented by high numbers of exploratory visits during acclimation (Figures 2A,B) especially in the first 24 h (5.45 ± 0.40, 2.03 ± 0.17 exploratory visits/h for the control and VPA group, respectively). The VPA rats showed a significant decrease in exploratory visits in the first 24 h (*p* < 0.001). Later, this initial exploratory activity decreased in the control group and showed a stable daily pattern (Figure 2A). The VPA group showed significantly lower initial exploratory activity in nose-poke learning (cosinor regression, difference in mesor, average activity; 33.87 vs. 22.94 visits/h for control and VPA group, respectively; *p* < 0.05). The reduction of exploratory visits in the VPA group was robust and highly significant (GLM, *p* < 0.001) throughout acclimation, nose-poke- and side preference learning phases (VPA group −55% vs. control; Figure 2B).

### Circadian Rhythm Disturbance in VPA Rats

The daily activity pattern was assessed during the acclimation phase when water was freely available. The circadian activity was estimated based on an analysis of daytime mean activity. The circadian rhythm of the VPA group showed an approximately 2-h shift after light off compared to the control group (*p* < 0.05; 7:00 for VPA compared to 5:01 for control; Figure 2C). Cry1, Per1, Arntl, Npas2, Clock and Mtnr1a gene expression was measured but did not reach a 2-fold change between the treatment groups, hence it cannot be interpreted as biologically relevant change. VPA group data are presented in the order of the following brain regions: cerebellum (C), HPC, prefrontal cortex (PFC) and thalamus (T).

Cry1 (C) 1.07, (HPC) 1.16, (PFC) 0.998, (T) 0.764; Per1 (C) 1.007, (HPC) 1.224, (PFC) 0.723, (T) 1.166; Arntl (C) 0.712, (HPC) 0.639, (PFC) 0.813, (T) 1.07; Npas2 (C) 0.717, (HPC) 0.924, (PFC) 0.828, (T) 1.126; Clock (C) 0.89, (HPC) 1.069, (PFC) 0.735, (T) 1.058; Mtnr1a (C) 0.697, (HPC) 0.886, (PFC) 1.497, (T) 0.706.

### Normal Place Preference and Reversal Learning in VPA Rats

Control and VPA groups did not differ in place and side preference learning abilities, nor was a difference in their reversal learning (Supplementary Figure S1). The proportion of correct nose-pokes was identified: 89.2% for the control and 88.1% for the VPA group (fitted value *χ*^2^ = 0.898, *p* = 0.3).

### Altered Drinking Behavior in VPA Rats

**Figure 3:**
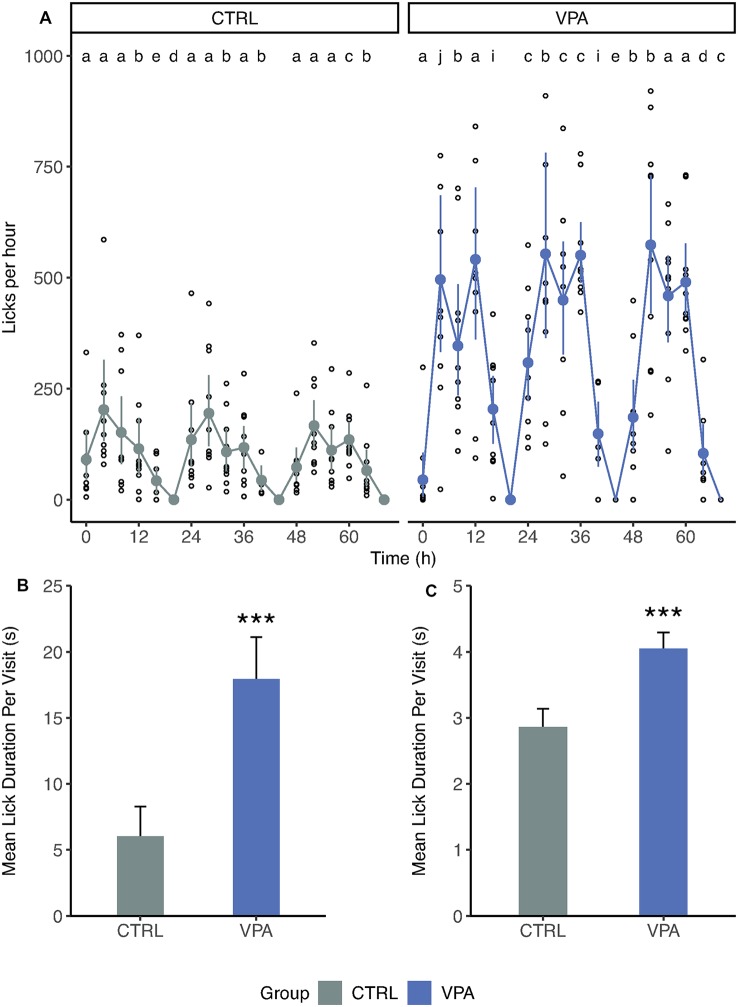
Differences in drinking behavior.** (A)** The number of licks grouped for 4-h periods during acclimation. Mean, confidence interval and individual values are shown. The number of lick values is transformed for 1 h. Letters indicate the individual with the highest number of licks for each 4-h period. **(B)** Mean lick duration (±SEM) per visit during acclimation (*p* < 0.001). **(C)** Mean lick duration (±SEM) per visit during later phases (pooled). ***p ≤ 0.001.

During acclimation, the daily pattern of drinking behavior showed frequent but short bouts for the control group, while VPA rats drank in rare but long bouts (Supplementary Figure S2). Consequently, control rats showed a well-balanced, stable pattern of lick number per hour distribution (Figure 3A). In the control group, 56% of the 4-h-long period was dominated by one subject (“a”) who drank the most during this time. In contrast, in the VPA group the individual animal with the highest number of licks (“a”) dominated only 24% of the 4 h periods.

Such distribution could arise from the differences in the number of visits or that of lick duration during visits. Therefore, we had investigated the mean lick duration per visit and found that there was an almost threefold difference between control and VPA rats for the mean lick duration during acclimation (6.0 s vs. 17.9 s linear mixed-effects model *p* < 0.001; Figure 3B). We pooled these data for nose-poke learning, side preference learning and competition, as well (Figure 3C). Although each protocol after acclimation was set up to limit lick duration to 7 s, there was a significant difference between groups (2.9 s vs. 4.1 s, linear mixed-effects model *p* < 0.001; Figure 3C). The sera of the groups were analyzed for blood chemistry parameters (Supplementary Figure S3, Supplementary Tables S1, S2). Out of 20 parameters only the potassium showed a slight increase compared to the reference values (5.29 for control and 4.64 for the VPA groups; reference values are for the potassium 4–5.9 mmol/l (Stender et al., 2007).

### Disturbed Hierarchy in VPA Rats

**Figure 4:**
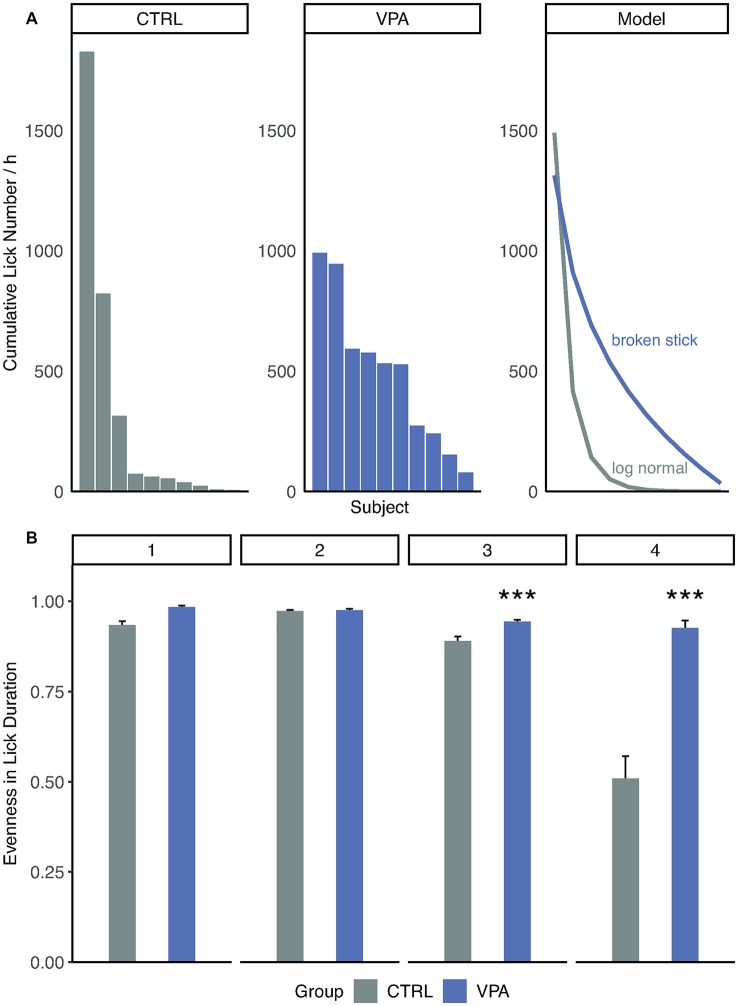
Inter-individual drinking behavior of treatment groups.** (A)** Distribution of cumulative lick number per hour. Rank abundance curves are drawn according to the model best fit the data: log-normal for control Akaike information criterion (AIC = 171.5) and broken stick for VPA group (AIC = 184.9). **(B)** Evenness (diversity divided by maximum diversity) values for groups and phases (daily mean ± SEM; ***p ≤ 0.001).

To reveal differences in group dynamics and adaptive behavior within the groups, a deeper analysis of drinking and visiting patterns was performed. During the competition phase of the study, three control subjects showed apparent competitive dominance over the rest of the animals which was manifested in the larger cumulative lick number (Figure 4A). In contrast, such a clear formation of subgroups was not observed within the VPA group (Figure 4A). When tested this in rank-distribution models, the best fit for the control group was log-normal (AIC = 171.5), whereas for the VPA group followed a broken-stick model (AIC = 184.9). This difference only appears when access to water is strictly limited in time and space. High evenness values were observed through acclimation and nose-poke learning phases, without significant difference between the groups (Figure 4B). As water became less accessible in, side preference learning and even more so in competition phases, the control group showed significantly lower evenness in lick duration, while in the VPA group it remained stable indicating an intensifying hierarchy for the control animals (Figure 4B, *p* < 0.001).

**Figure 5:**
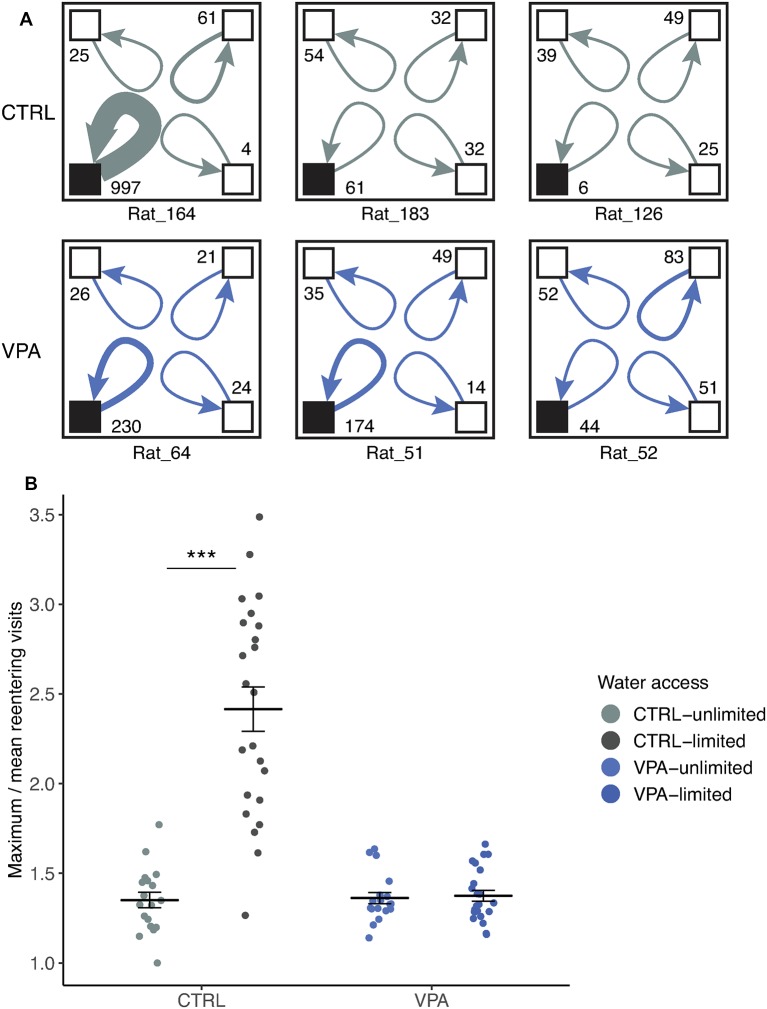
Lack of social dominance in the VPA group. **(A)** Representative cage layouts for three individuals (ID presented at the bottom of the schematic cage) with the highest, median and lowest numbers of reentering visits for each treatment group (rows) during competition. Reentering visits are calculated for each subject as visits to the same corner when the subject left without other subjects entering the corner (numbers shown at the corners). The thickness of the arrows indicates the strength of the reentering visits. **(B)** Social dominance within the group is characterized by using a ratio of the maximum visits of an individual divided by the mean values of the group (±SEM) for daily cumulative reentering visits. Water limitation provoked the elevation of social dominance in the control group as compared to conditions with unlimited water access (effect size: 78.8%, ***p ≤ 0.001), whereas no significant effect was shown within the VPA group that remained at a dominance value below 2.

Representative subjects’ with most, median and least number of consecutive visits are shown to the same corner during the competition (Figure 5A). In the control group, one animal had a substantially higher number of consecutive visits compared to the rest. VPA group’s subjects showed much more converging numbers. The entire population was thus described using maximum over mean consecutive visits calculated for each day of the experiment.

There was a significant difference in the limited or unlimited conditions of water access within the control group (Figure 5B) when the daily maximum/mean reentering visits (i.e subject reentering the same corner) were calculated. Control rats visited the same corner 78% more frequently when water access was scarce (competition phase), compared to the water unlimited condition (*p* < 0.001). In contrast, the limitation of water access did not change the behavior of VPA rats; they did not change the frequency to reenter the same corner in any condition (Figure 5B).

## Discussion

Collecting very detailed information in a social environment may provide novel insights on fundamental elements of behavior that cannot be deduced from traditional behavioral observations that deliver only limited amounts of data. In the present study, data mining of the pattern of simple animal actions; visiting, nose-poking and drinking allowed us to investigate various behaviors in communities of prenatally VPA-exposed autistic-like and control rats. The autistic behavior of rats prenatally treated with VPA includes social components resembling the symptoms of human ASD (Schneider and Przewłocki, 2005). Here, we focused on more sophisticated aspects of behavior beyond the well-known social effects described in VPA animals.

VPA-treated rats showed a drastic decrease in exploratory visits in the first 24 h compared to the control group, which was maintained at some level in all phases except competition. One can speculate, that the reason behind decreased exploratory behavior during competition is that both groups optimized their behavior in a way that they did not “waste” visits only for exploration and took every chance to drink whenever possible. The overall decrease of exploration may derive from neophobia, anxiety or alternatively, a shifted circadian rhythm could also play a role.

Indeed, our results showed a 2-h shift in the circadian rhythm of the VPA group. In parallel with our result, an earlier report showed that VPA shortened circadian period *in vivo* and *in vitro* and suppressed behavioral activity across species (Landgraf et al., 2016). ASD patients often have sleep problems and altered social timing, and it has been correlated with identified single nucleotide polymorphisms (SNPs) of Per1 and Npas2 genes (Nicholas et al., 2007). VPA exposure alters core circadian rhythm transcription factors *in vitro* and *in vivo* (Olde Loohuis et al., 2017; Griggs et al., 2018). In both studies, altered expression of known circadian rhythm genes was reported. Interestingly, our results showed no significant change in a selected set of circadian rhythm genes (Cry1, Per1, Arntl, Npas2, Clock and Mtnr1a) of the VPA rats. This may suggest that our behavioral findings in the VPA group are not the consequence of the differentially expressed mRNA levels of the circadian genes, rather on the level of proteins, SNPs or changed regulation of certain transcription factors. On the other hand, considering the highly complex and multifactorial nature of the circadian regulation, we only analyzed a small set of genes, hence we must be careful when drawing conclusions. Circadian changes due to different patterns of melatonin expression were studied in human ASD patients (Nir et al., 1995). In parallel with these studies, our results confirm the circadian rhythm disturbance *in vivo*.

During the acclimation phase, we have unexpectedly found a substantial difference in general drinking behavior of the VPA group that appeared as an almost threefold increase in lick duration per visit. Control rats tended to visit very often but drank only in short bouts while VPA rats visited rarely but once they entered the corner, they drank considerably more. In the following phases, this difference in behavior stayed significant despite the limitation of water access (7 s). Altered drinking behavior was most likely not due to any underlying mechanism of the VPA treatment, as we did not see major anomalies in blood chemistry results (Supplementary Figure S3, Supplementary Table S1). Only the potassium level showed a slight increase in the control group compared to the upper limit of the reference value. However, the extent of the difference seems irrelevant (0.49 mmol/l) that could be only a consequence of the higher water intake, as well. Therefore, we concluded, that there was no apparent water balance issue that could explain the high duration of licks. This altered drinking behavior in VPA rats could be explained by increased perseveration (represented by mean lick duration increase) that might result from the prenatal VPA exposure. The increase in water consumption could be also explained by psychogenic polydipsia (PPD). PPD is a clinical symptom, which has a common occurrence in patients with psychiatric disorders, most commonly schizophrenia (de Leon et al., 1994). PPD is characterized by polyuria and polydipsia (Dundas et al., 2007). We believe that this is the first preclinical study showing the appearance of PPD in conditions that can be associated with human ASD. This phenomenon was already shown in autistic children, which further strengthens the translational value of the prenatal VPA rat model of ASD (Terai et al., 1999).

Puścian et al. (2014) reported earlier impaired place preference learning of prenatally VPA treated C57BL/6 and BALB/c mice using automated home cages, while we found no difference in side preference and reversal learning of VPA rats. Because of the difference in spatial learning paradigms between the two studies, it is difficult to compare results. The differing results of the two studies may be explained by the application of different species (mice vs. rats).

The use of the automated home cage for behavioral experiments has the remarkable potential to reveal the naturally occurring spontaneous hierarchy and social structure within the small rat population of the cage. The interaction of multiple social and cognitive skills is needed to organize a functioning hierarchy that in nature increases the chance of survival of a population in gregarious species. It has been even suggested that social organization is one of the biggest evolutionary driver of mammals (Christian, 1970). We hypothesized that impaired social and communicative skills typical of the VPA model can result in unstable or disorganized hierarchy of a group with autistic-like symptoms. The IntelliCage allows us to examine not only the behavior of individual rats but also enables us to analyze the inter-individual interactions that can lead to a better understanding of how a group behaves in certain situations. We designed a competitive task in which water availability was spatially and temporally limited. Spontaneous hierarchy and its alteration in a highly competitive task were assessed using rank abundance curves and evenness. During the competition, hierarchy in the control group is characterized by unevenness and log-normal distribution of lick duration, both features lacking in the VPA group. Supporting our results, a recent study reported disturbed hierarchy in Fmr1 KO rats, revealed by dominance tube tests (Saxena et al., 2018). Fmr1 KO is a model of Fragile X syndrome, frequently co-diagnosed with ASD.

In a competitive situation, subjects have essentially two distinct ways of keeping others from drinking: repelling others and occupying or guarding the rewarded corner. In the first situation, inter-visit intervals would be higher after dominant subjects exit the corner compared to subordinate animals. However, this was not the case: the distribution of inter-visit intervals did not depend on subjects (data not shown). On the other hand, guarding behavior would increase the number of reentering visits by dominant subjects. Our study revealed a guarding behavior of the control rats, represented by the increased reentering visit number when water was limited. Strict hierarchy and guarding the water source was not necessary when it was more available. In contrast, guarding behavior did not evolve in VPA rats when water became limited. Therefore, VPA rats could not develop stronger hierarchy upon water scarcity. The most striking discovery of the present study is that failure in changing to uneven resource sharing and lack of guarding can be interpreted as social/supra-individual rigidity in VPA rats. The occurrence of unevenness (representing the strength of hierarchy) was already apparent in, side preference learning but even more pronounced during competition. We suspect that in control rat’s strength of hierarchy is inversely proportional with water availability. They were able to adapt to the changing environment by changing their behavior.

As resource distribution becomes patchy, the members of a group are forced into more direct competition with each other for better utilization of each patch of resource (Meurant, 1988). In nature, more subordinate individuals will leave the group or will not survive (Meurant, 1988). To put differently, when resources are scarce, uneven resource sharing serves the survival of the fittest. Limiting a vital resource, water and studying its utilization among the group members gave us an insight into the altered social economy of autistic-like rats.

On the contrary, the VPA group did not adapt to decreasing resources by uneven sharing. This maladaptive behavior could be strongly correlated with behavioral or cognitive rigidity as reported earlier in prenatally VPA treated mice (Puścian et al., 2014). Cognitive rigidity is a known hallmark of autistic-like traits on the level of individual subjects, as well (Karvat and Kimchi, 2014). Even though “awkwardness” in social situations, struggling to recognize social rank in society are considered core symptoms of ASD, there is very limited scientific literature covering the topic of social dominance in ASD. However, a recent study shows that subjects living with ASD tend to judge dominance in a social interaction slower, indicating malfunctioning nonverbal processing (Kuschefski et al., 2019). There are some other indirect examples which stem from dysfunctional social hierarchy recognition in ASD. Sterzing et al. (2012) found that children with ASD are bullied nearly five times more often than their neuro-typical schoolmates. This latter finding may derive from the fact that children with ASD are less able to acknowledge or respect someone with higher social rank in their community. Taken these results together with our findings, the disrupted dominance hierarchy among autistic rats can be related to clinical manifestation.

Our approach of investigating the social and non-social behaviors of VPA-treated rats in automated home cages led us to detect novel characteristics of the prenatal VPA rat model of ASD in an etiologically more relevant design. Our results showed PPD, decreased exploration and altered circadian rhythm of autistic rats. The most salient finding is that prenatally VPA-exposed rats as a group show an inability to adapt their behavior in a changing environment. Since substantial impairments of adaptive behavior are a hallmark feature of ASD with serious consequences on the everyday functioning of individuals, this finding further increases the translational value of the prenatal VPA model and may indicate its potential usefulness in ASD drug discovery.

## Data Availability Statement

The datasets generated for this study are available on request to the corresponding author.

## Ethics Statement

The animal study was reviewed and approved by Local Animal Care and Use Committee (PE/EA/2885-6/2016) and were carried out in accordance with European Animal Protection Directives (Directive 2010/63/EU).

## Author Contributions

PP, BL, VR and GL conceived the study and wrote the manuscript. PP and GN implemented the experiments. PP and KK analyzed the data and participated in the interpretation of the results. CC participated in the experimental design and conceiving the study. All authors read and approved the final version of the article.

## Conflict of Interest

All authors were employed by the company Gedeon Richter Plc.

## Footnotes

^1^https://www.tse-systems.com/product-details/intellicage

^2^https://www.R-project.org/
