# Supplementary material for: Disrupted Social Hierarchy in Prenatally Valproate-Exposed Autistic-Like Rats
Source: Front Behav Neurosci. 2020 Jan 15;13:295. doi: 10.3389/fnbeh.2019.00295 (PMC6974458; doi:10.3389/fnbeh.2019.00295)
Supplement: Supplementary file 1 [file Data_Sheet_1.docx]

Supplementary Material

# Supplementary Data

Control and VPA groups did not differ in place and side preference learning abilities, nor was difference in reversal learning (Supplementary Figure 1). Proportion of correct nosepokes was 89.2% for the control and 88.1% for the VPA group (fitted value Chi2=0.898, p=0.3). During acclimation, the daily pattern of drinking behavior showed frequent but short bouts for the control group, while VPA rats drank in rare but long bouts (Supplementary Figure 2D). Sera of the groups were analyzed for blood chemistry parameters (Supplementary Figure 3, Supplementary Table 1 and 2). Out of 20 parameters only the potassium showed a slight increase compared to the reference values (5.29 for control and 4.64 for the VPA groups; reference values are for the potassium 4.08-4.8 mmol/liter). Autistic behavior phenotype was characterized by a series of assays before the beginning of the IntelliCage study (Supplementary Figure 4). Juvenile social play, spontaneous locomotor activity, ultrasonic vocalization and von Frey test were carried out. VPA rats showed a non-significant decrease in pinning numbers (21.3±2.343 vs. 15±3.639 n=10,10). In spontaneous locomotor activity test ambulation did not differ significantly between the groups (593.4±57.91 vs. 553.7±34.83). VPA rats showed a significantly lower rearing count compared to the control group (101.7±7.765 vs. 64±5.348 n=10,10; p=0.0008). The number of calls in ultrasonic vocalization test was also significantly lower in VPA group (396.4±139.2 vs. 23.2±11.31 n=10,10; p=0.0155). The threshold was significantly lower of the VPA group (23.2±1.489 vs. 14±1.506 n=10,10; p=0.0004) in the von Frey test.

## Supplementary Figures and Tables


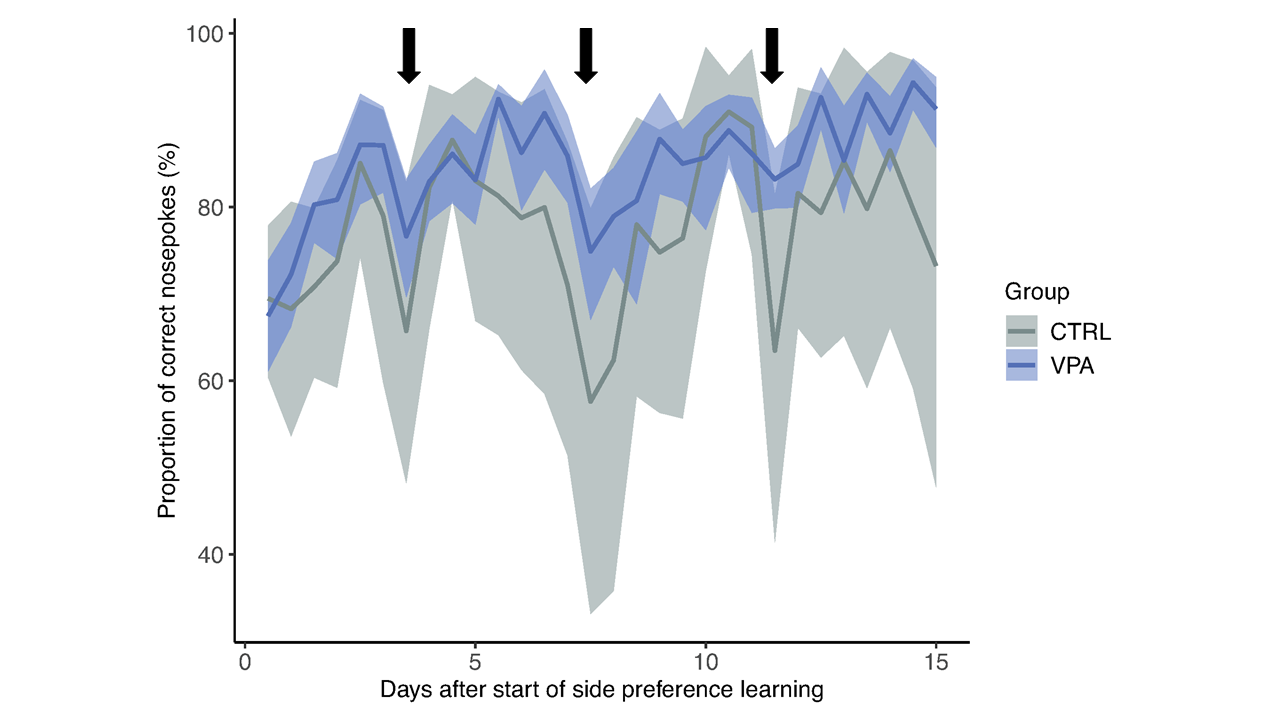


**Supplementary Figure 1.** Side preference learning of groups. Proportion of correct nosepokes to all nosepokes is expressed as percentage. Arrows indicate side reversals. Lines denote mean, while ribbons show confidence interval for treatment groups.


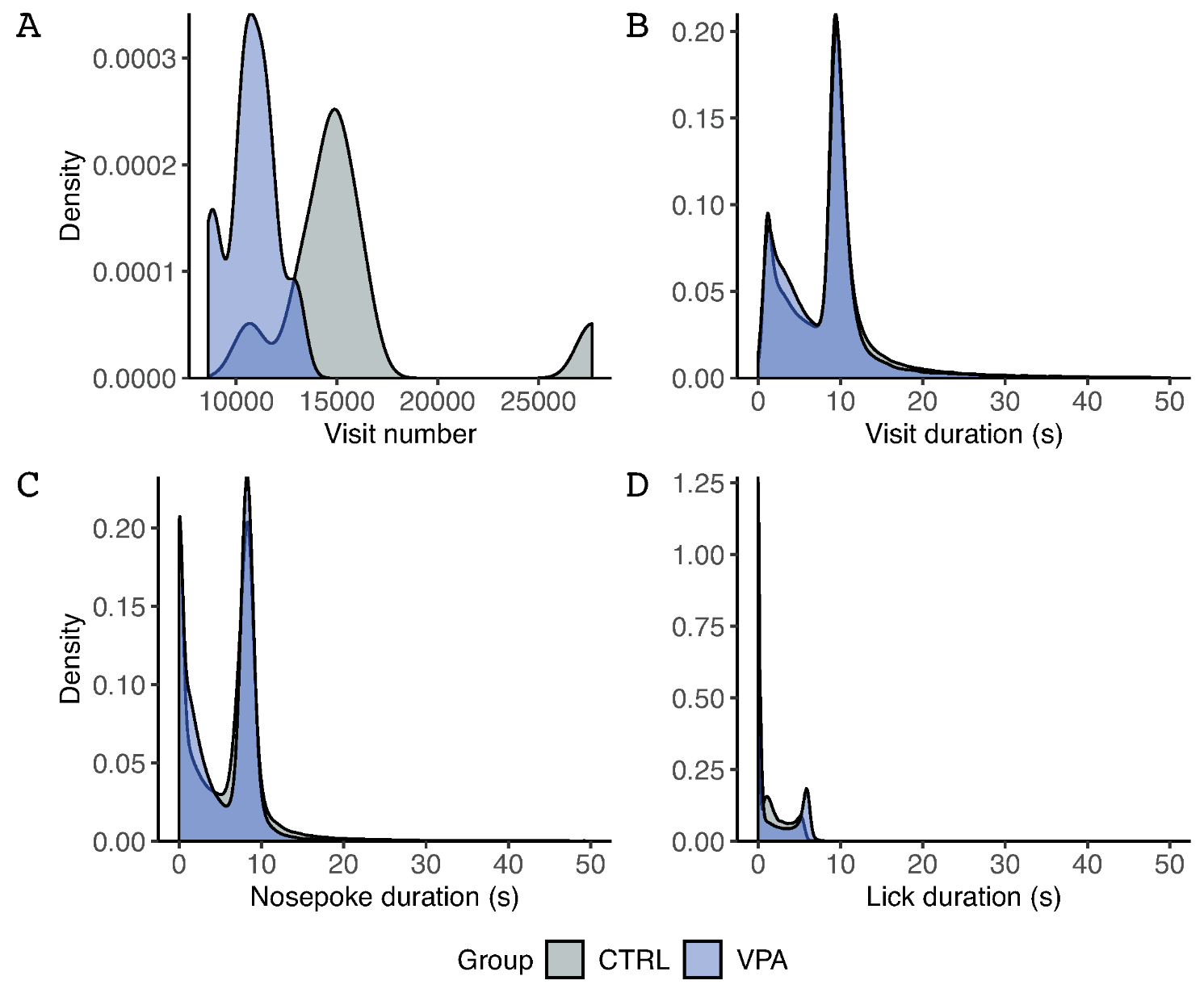


**Supplementary Figure 2.** Descriptive density plots for (A.) visit number, (B.) visit duration, (C.) nosepoke duration and (D.) lick duration


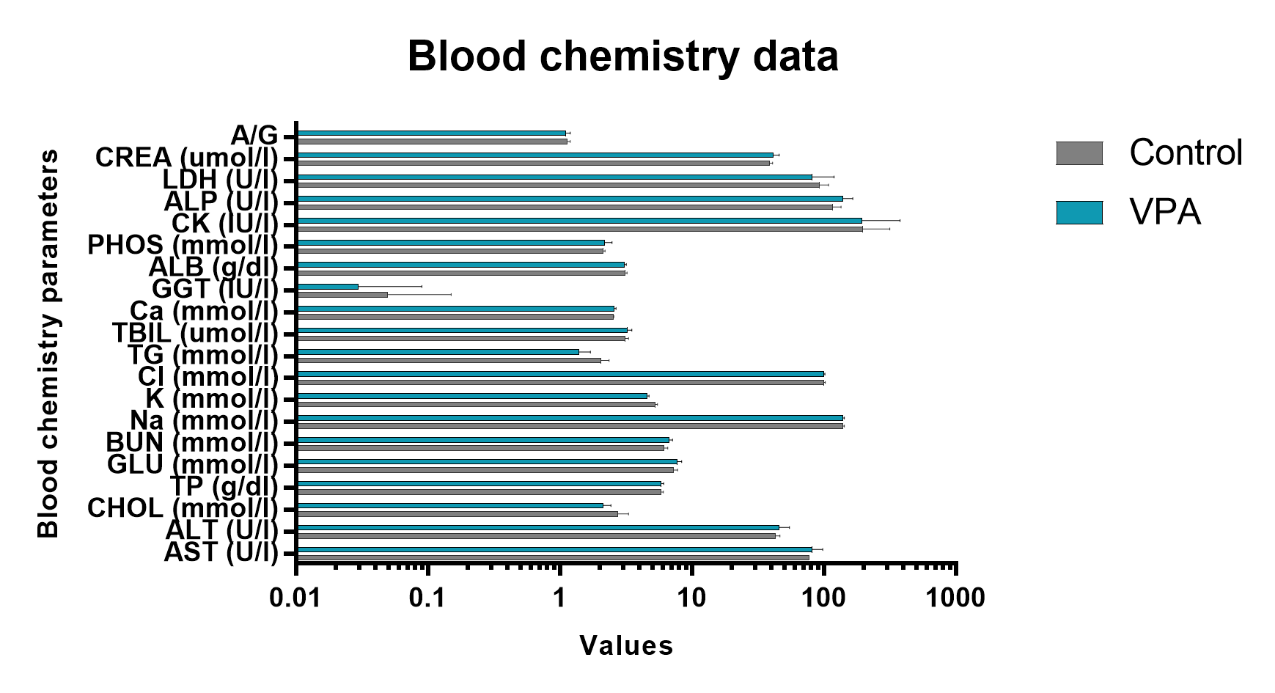


**Supplementary Figure 3.** Blood chemistry parameters of treatment groups. Means (±SD) are shown.


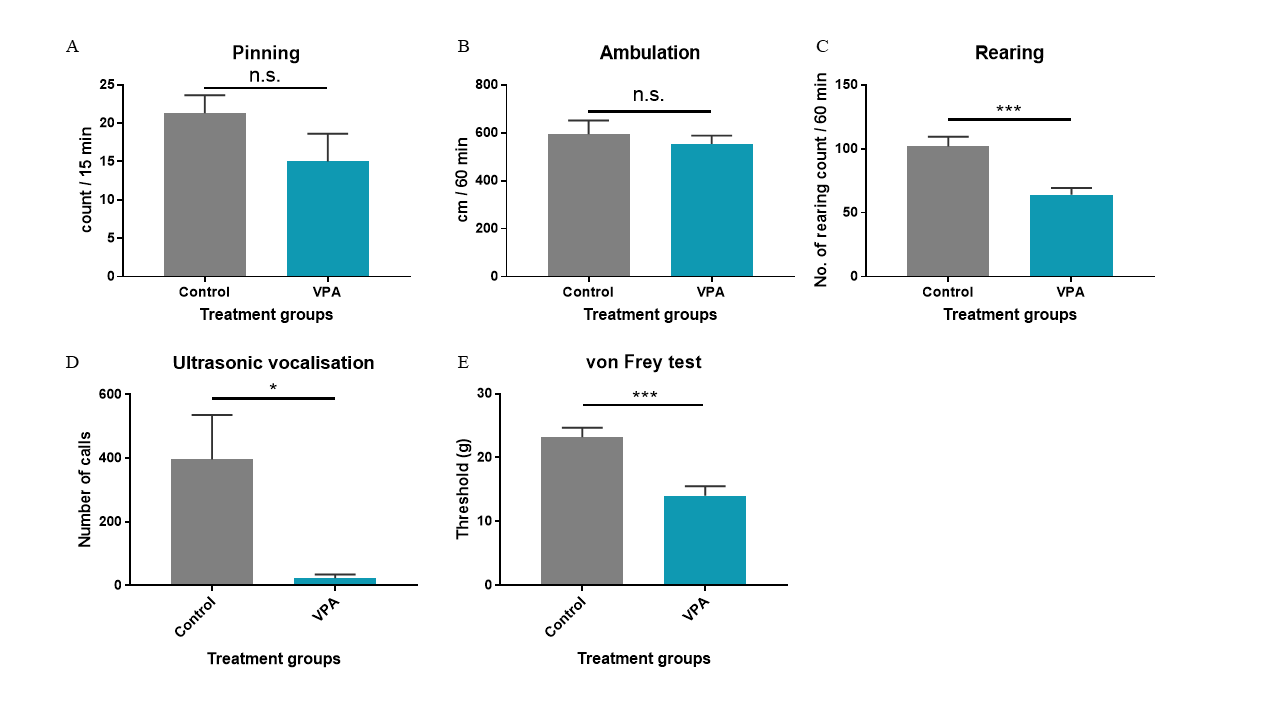


**Supplementary Figure 4.** Summary of phenotype characterization tests on control and VPA rats. Means (±SEM) are shown.


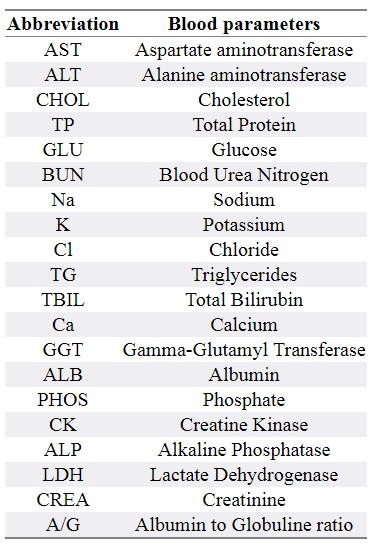


**Supplementary Table 1.** Abbreviations of the blood chemistry parameters.

|  | Control | | | VPA | | |
| --- | --- | --- | --- | --- | --- | --- |
| parameters | mean | SD | n | mean | SD | n |
| AST (U/l) | 77.8 | 6.9 | 6 | 82.4 | 15.7 | 5 |
| ALT (U/l) | 43 | 3.69 | 6 | 46 | 8.89 | 5 |
| CHOL (mmol/l) | 2.75 | 0.56 | 6 | 2.13 | 0.31 | 5 |
| TP (g/dl) | 5.9 | 0.19 | 6 | 5.9 | 0.24 | 5 |
| GLU (mmol/l) | 7.34 | 0.5 | 6 | 7.82 | 0.56 | 5 |
| BUN (mmol/l) | 6.18 | 0.38 | 6 | 6.78 | 0.39 | 5 |
| Na (mmol/l) | 141 | 2.71 | 6 | 140 | 3.42 | 5 |
| K (mmol/l) | 5.29 | 0.22 | 6 | 4.64 | 0.1 | 5 |
| Cl (mmol/l) | 101 | 1.63 | 6 | 100 | 2.19 | 5 |
| TG (mmol/l) | 2.06 | 0.31 | 6 | 1.41 | 0.3 | 5 |
| TBIL (umol/l) | 3.14 | 0.17 | 6 | 3.28 | 0.24 | 4 |
| Ca (mmol/l) | 2.55 | 0.02 | 6 | 2.58 | 0.09 | 5 |
| GGT (IU/l) | 0.05 | 0.1 | 6 | 0.03 | 0.06 | 5 |
| ALB (g/dl) | 3.15 | 0.08 | 6 | 3.11 | 0.08 | 5 |
| PHOS (mmol/l) | 2.13 | 0.07 | 3 | 2.19 | 0.29 | 5 |
| CK (IU/l) | 199 | 118 | 6 | 196 | 183 | 4 |
| ALP (U/l) | 118 | 17.7 | 6 | 140 | 25.9 | 5 |
| LDH (U/l) | 93.8 | 15.2 | 4 | 82.4 | 37.8 | 5 |
| CREA (umol/l) | 38.9 | 1.95 | 6 | 41.5 | 4.49 | 5 |
| A/G | 1.15 | 0.05 | 6 | 1.12 | 0.08 | 5 |

**Supplementary Table 2** Descriptive data of blood parameters.
